# Pneumomediastinum, pneumopericardium, and subcutaneous emphysema—a rare complication in COVID-19 infection

**DOI:** 10.1186/s43168-021-00081-y

**Published:** 2021-07-29

**Authors:** Archana Baburao, Rinki Das, Shylaja Shyamsunder

**Affiliations:** 1 Department of Pulmonary Medicine, Rajarajeswari Medical College and Hospital, Bengaluru, India; 2 Department of Pulmonary Medicine, Bhagawan Mahaveer Jain Hospital, Girinagar, Bangalore, India; 3 Department of Critical Care and Emergency Medicine, Bhagawan Mahaveer Jain Hospital, Girinagar, Bangalore, India; 4 Department of General Medicine, Bhagawan Mahaveer Jain Hospital, Girinagar, Bangalore, India

**Keywords:** Case report, Pneumomediastinum, Subcutaneous emphysema, COVID-19

## Abstract

**Background:**

Coronavirus disease 2019 (COVID-19) has become a global pandemic and is posing a serious public health problem for almost all countries. Spontaneous pneumomediastinum, a rare condition, is usually seen in patients with underlying pulmonary pathology, infections, or mechanical ventilation. Spontaneous pneumomediastinum is a rare complication in COVID-19 pneumonia.

**Case presentation:**

We report a case of spontaneous pneumomediastinum, pneumopericardium, and subcutaneous emphysema in a 62-year-old diabetic patient with COVID-19 infection who presented with cough, fever, and breathlessness, which turned to be a fatal complication.

**Conclusion:**

Pneumomediastinum/subcutaneous emphysema, a not so common complication associated with COVID-19 infection, should be considered as a bad prognostic indicator of worsening disease and hence requires early recognition and careful monitoring of the patient for any possible unfavorable outcome.

## Background

World Health Organization (WHO) declared COVID-19 as a global pandemic in March 2020 with more than ten million infections occurring in India. Many organs have been affected by this virus including cardiovascular, respiratory, gastrointestinal, neurological, hematopoietic, and immune system [1]. The disease has a diverse course ranging from asymptomatic infection to those with respiratory failure, complicated by acute respiratory distress syndrome (ARDS). Spontaneous pneumomediastinum is a rare complication in COVID-19 pneumonia [2]. We report a case of spontaneous pneumomediastinum, pneumopericardium, and subcutaneous emphysema in a COVID-19-infected patient.

## Case presentation

**Fig. 1:**
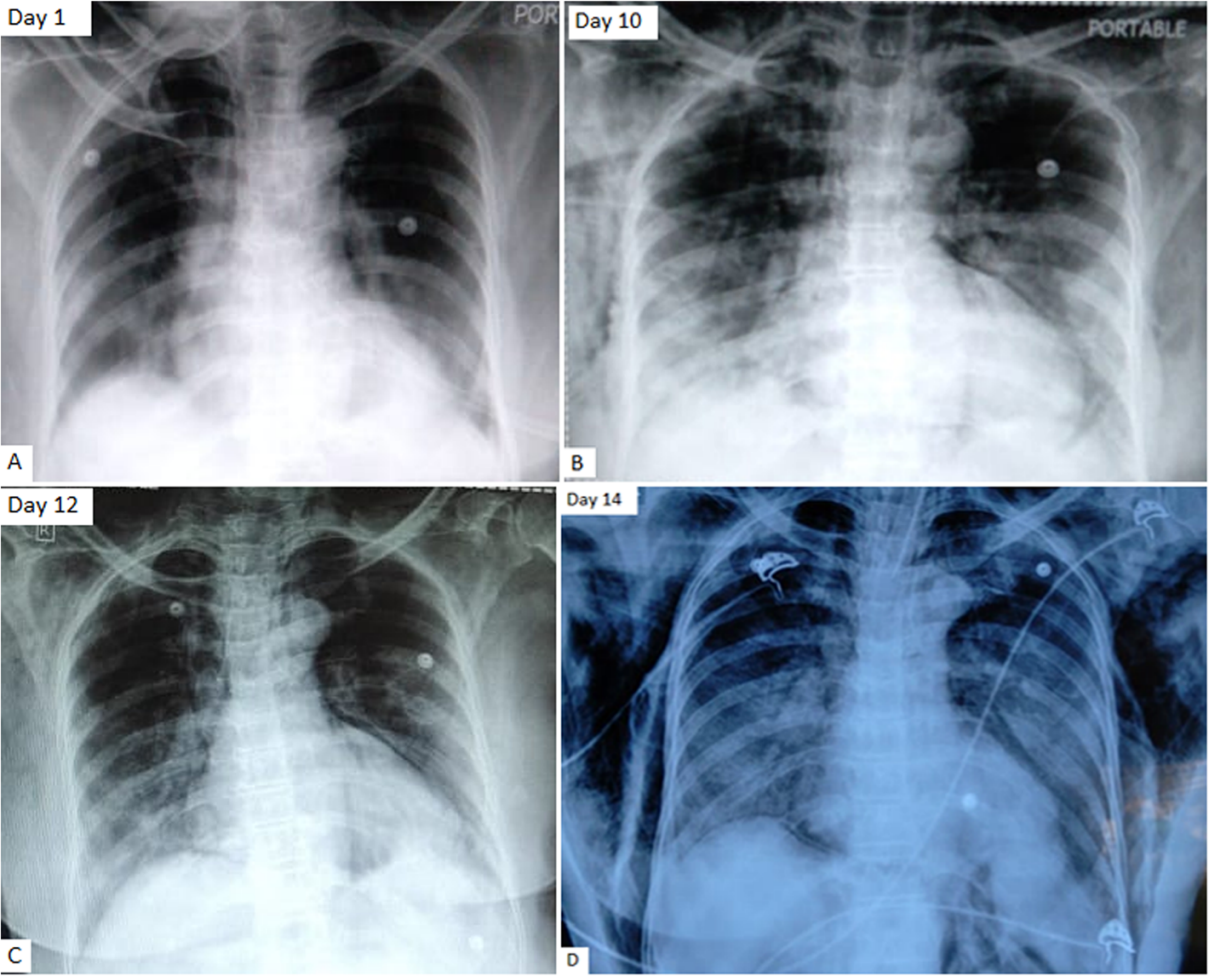
**A** Day 1 chest X-ray antero-posterior view showing bilateral pneumonia. **B** Day 10 chest X-ray showing subcutaneous emphysema and pneumopericardium. **C** Day 12 chest X-ray partial resolution of subcutaneous emphysema. **D** Day 14 chest X-ray showing recurrence of subcutaneous emphysema with an increase in lung shadows

**Fig. 2:**
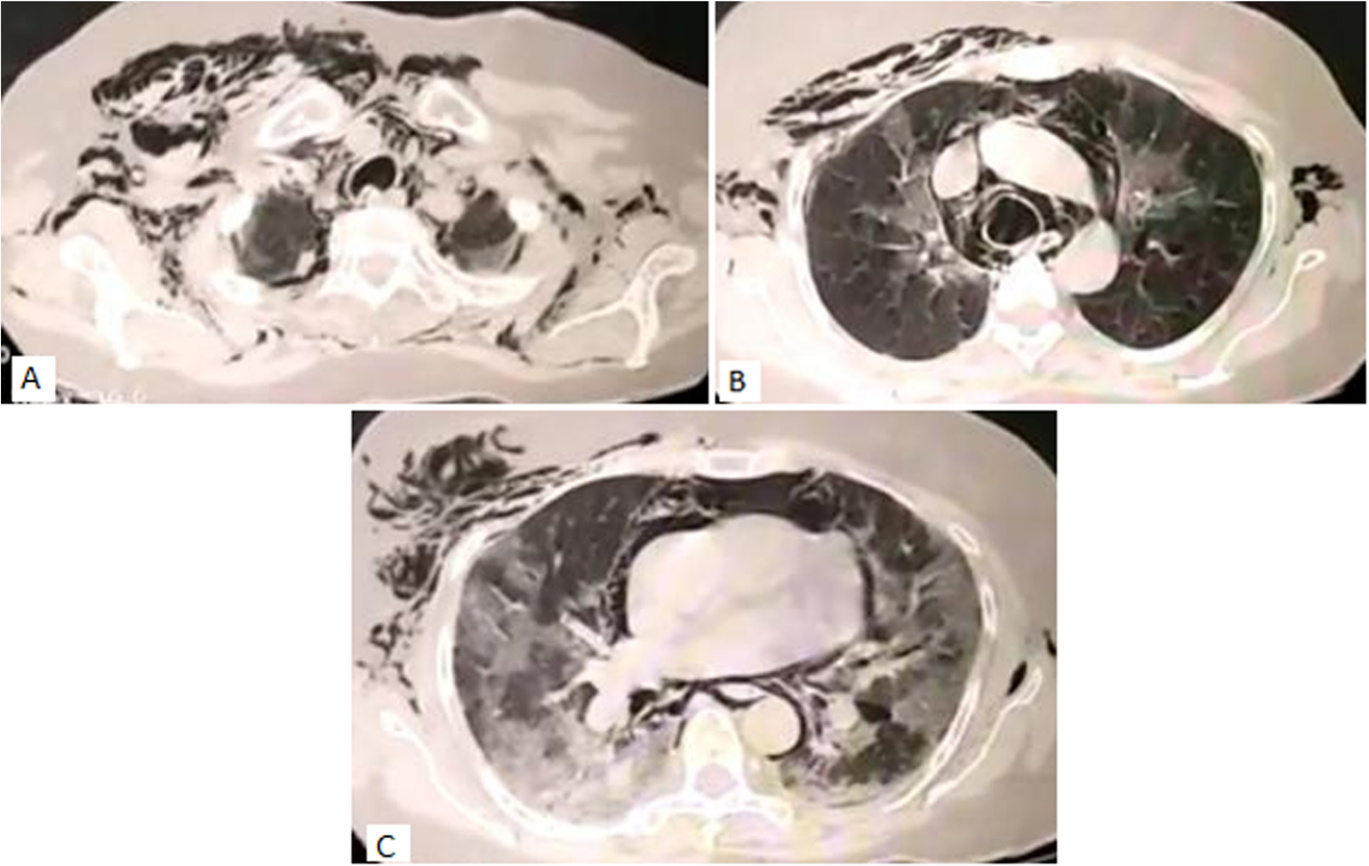
Axial CT chest images showing. **A** Subcutaneous emphysema extending up to posterior chest wall. **B** Presence of air around the trachea and major blood vessels indicative of pneumoediastinum along with bilateral upper lobe ground glass opacities. **C** Presence of air dissecting the pericardium indicative of pneumopericardium with bilateral extensive lower lobe ground glass opacities

A 62-year-old female presented with cough, fever, and breathlessness on exertion since 2 days. She was diabetic on oral hypoglycemic agents. She was not addicted to tobacco, alcohol, and illicit drugs and had no history of respiratory complaints in the past. However, she was the primary contact of a COVID-19-positive patient. On examination, she was conscious, oriented, febrile, and had tachycardia and tachypnea with a respiratory rate of 28/min and saturation of 86% at room air. Examination of the respiratory system revealed bilateral infrascapular crepitations. The rest of the physical examination was unremarkable. Due to the patients’ contact history and her symptom profile, reverse transcriptase polymerase chain reaction for COVID-19 was sent which tested positive. Arterial blood gas analysis showed type 1 respiratory failure with Pao_2_ 55 mmHg. Chest X-ray (CXR) showed bilateral mid and lower zone haziness with peripheral predominance (Fig. 1A). Complete hemogram revealed leukocytosis (13,100 cell/cumm) with neutrophilia (91.2%), lymphopenia (5.5%), and eosinopenia (0%). Liver function test was normal expect for mildly elevated enzymes (SGOT 75, SGPT 59). Her renal function, serum electrolytes, and coagulation profile were normal. Inflammatory markers were done which showed serum ferritin 178.27 ng/ml, C reactive protein 14 mg/L, d dimer 189 ng/ml, and serum lactate dehydrogenase 340 U/L. She was diagnosed as severe COVID-19 infection and started on IV remdesvir, IV steroids (methylprednisolone 0.75 mg/kg/day for 7 days), anticoagulants, IV piperacillin and tazobactum, supplemental oxygen through a non-rebreather mask, and other supportive treatments. Awake proning was encouraged and she received chest physiotherapy. Though she responded to the initial treatment, her oxygen requirement started increasing from day 8, and repeat inflammatory markers showed increasing trend serum ferritin 391.57 ng/ml, CRP 120 mg/L, D dimer 1017 ng/ml, and serum LDH 669 U/L with leucocyte count of 20,260 cell/cumm. However, her IL 6 was normal (20 pg/ml). Her antibiotic was escalated to IV meropenem and was put on a high flow nasal cannula (flow was initiated at 50–60 L/min with FiO_2_ 60–70%, titrated to aim for an oxygen saturation ≥ 92%). She also received two units of plasma therapy. On day 10, she developed bilateral swelling over the chest with palpable crepitus. Repeat CXR showed radiological worsening of lung shadows with air in the subcutaneous tissue (Fig. 1B). CT thorax showed subcutaneous emphysema with pneumomediastinum, pneumopericardium (Fig. 2A–C) without pneumothorax which was managed with continuing the supplemental oxygen therapy. Even though there was an initial reduction in subcutaneous emphysema and pneumomediastinum (Fig. 1C), the patient became increasingly hypoxic with recurrence of both with worsening of lung shadows (Fig. 1D) for which she was intubated on day 14 and connected to a mechanical ventilator with lung protective criteria (lower tidal volumes 6 ml/kg of ideal body weight with plateau pressure < 30 cmH2O). Her repeat leucocyte count was persistently high for which antibiotic was further escalated to IV colistimethate sodium. She also required vasopressor support on day 15 and developed multi-organ failure succumbing to the infection on day 16.

## Discussion

COVID-19 is an ongoing global pandemic which has rapidly spread over several months, affecting patients across all age groups and geographic areas. It has become a major health problem causing severe acute respiratory illness in humans. Pulmonary symptoms and signs dominate the clinical picture and CXR and chest CT play an important role in diagnosis and assessing the progression in these patients. CXR predominantly shows bilateral disease with a tendency toward the lung periphery and Chest CT images are most notable for showing bilateral ground glass opacities and consolidation mainly in peripheral/subpleural region with predominant involvement of lower lung lobes and posterior segments with the absence of concomitant pulmonary nodules, cavitation, adenopathy, and pleural effusions [3, 4].

Pneumomediastinum is the presence of extra-alveolar air in the mediastinum. It can be spontaneous from a predisposing factor or secondary to underlying pulmonary cause and COVID-19 is a new addition to it, which is rarely reported in literature. The pathophysiological process as described by Macklin involves alveolar rupture, air dissection along bronchovascular sheaths, and spread of this blunt pulmonary interstitial emphysema into the mediastinum. The rupture along the alveolar tree releases alveolar air which centripetally dissects through the pulmonary interstitium along the bronchovascular sheaths toward the pulmonary hila [5].

SARS-CoV-2 (severe acute respiratory syndrome coronavirus 2) preferentially infects type II pneumocytes resulting in diffuse alveolar damage with fibrin rich hyaline membrane formation consistent with ARDS [6, 7]. This damage to the integrity of the alveolar membrane causes pressure gradient difference between alveoli and lung interstitial tissue resulting in spontaneous pneumomediastinum.

Lemmers et al. demonstrated a sevenfold increase in the development of pneumomediastinum and/subcutaneous emphysema in patients with ARDS and COVID-19 compared to patients with ARDS from other causes, despite employing a protective mechanical ventilation strategy and they attributed it to the increased lung fragility and lower compliance of the respiratory system in COVID-19 associated ARDS than barotrauma [8]. Whenever barotrauma is excluded, the underlying disease should be considered as the cause for the pneumomediastinum/subcutaneous emphysema. Although pneumomediastinum is usually considered a self-limiting condition which responds favorably to conservative therapeutic measures, it can progress to life-threatening cardiovascular and respiratory complications [9], as we encountered in our patient in terms of increasing hypoxia and need for ionotrope support. The development of pnemomediastinum in COVID-19 pneumonia is considered a bad prognostic indicator of worsening disease indicating severe damage to the alveolar membrane [10]. In our patient too, initially, pnemomediastinum/subcutaneous emphysema responded to conservative management but it recurred with worsening hypoxia indicating disease progression. Hence, the presence of pneumomediastinum early in the course of illness should alert the possibility of underlying disease progression and the need for close monitoring.

## Conclusion

Spontaneous pneumomediastinum is a serious complication in COVID-19 pneumonia and requires early recognition. Through this case report, we wanted to describe this complication as a potential indicator of worsening disease.

## Data Availability

Available

## Abbreviations

WHO: World Health Organization
ARDS: Acute respiratory distress syndrome
RTPCR: Reverse transcriptase polymerase chain reaction
LFT: Liver function test
RFT: Renal function test
CXR: Chest X-ray
SGOT: Serum glutamic oxaloacetic transaminase
SGPT: Serum glutamic pyruvic transaminase
CRP: C reactive protein
LDH: Lactate dehydrogenase
CT: Computer tomography
SARS-CoV-2: Severe acute respiratory syndrome coronavirus 2
